# Uterine rupture during labor in a patient with endometriosis: A case report

**DOI:** 10.1016/j.crwh.2026.e00789

**Published:** 2026-02-08

**Authors:** Julia Häusler, Claudia Studer, Kathrin Bütikofer, Nebojša Stevanović

**Affiliations:** a Department of Obstetrics and Gynecology, Cantonal Hospital Olten, Baslerstrasse 150, 4600 Olten, Switzerland; b University of Basel, Petersplatz 1, 4001 Basel, Switzerland

**Keywords:** Uterine rupture, Adenomyosis, Endometriosis, Case report

## Abstract

Uterine rupture is a rare obstetric complication, particularly in women without a prior cesarean delivery. This report describes an intrapartum posterior uterine wall rupture extending into the vagina in a patient with pre-existing endometriosis and presumed adenomyosis, highlighting endometriosis and its surgical management as potential risk factors. A term gravida with a history of two laparoscopic procedures for endometriosis presented with menstrual-like bleeding and contractions. During labor, she developed severe abdominal pain, prompting an emergency cesarean section. Intraoperatively, a 12 cm longitudinal rupture of the posterior uterine wall extending into the pouch of Douglas and the posterior vaginal fornix was identified. The defect was successfully sutured, and hysterectomy was not required. The postpartum outcome for both the mother and the newborn was satisfactory. This case underscores the importance of recognizing endometriosis and its surgical treatment as possible contributing factors to uterine rupture.

## Highlights

• A rare case is reported of posterior uterine rupture in labor without prior cesarean delivery.
• Th patient had endometriosis, for which she had had prior laparoscopic surgical treatment.
• Emergency cesarean section revealed a 12 cm posterior wall rupture into the vagina.
• No hysterectomy was required, and both mother and child had a favorable outcome.
• Endometriosis and its surgery may be underrecognized risk factors for rupture.

## 1 Introduction

Uterine rupture is a rare obstetric complication but carries a high risk of maternal and fetal mortality [1]. Established risk factors include prior cesarean delivery or surgical myomectomy [2], [3], [4]. In the absence of a previous cesarean section, uterine rupture is exceedingly uncommon, with an estimated incidence of less than 1 in 10,000 women [5]. Because of its rarity in such cases, the underlying risk factors remain poorly understood. This report describes a case of spontaneous longitudinal rupture of the posterior uterine wall extending to the vagina, which occurred in a patient with pre-existing endometriosis. It highlights endometriosis and/or its surgical management as possible risk factors for uterine rupture.

## 2 Case Presentation

A 38-year-old woman (gravida 1) presented at 40 weeks of gestation with menstrual-like vaginal bleeding, rupture of membranes, and regular contractions, with a cervical dilation of 1 cm. Upon admission, no cause for the bleeding could be identified. Ultrasound showed no signs of placental abruption, fetal hemoglobin in maternal blood was not elevated, and the fetal heart rate was normal.

The patient had a five-year history of infertility, but had conceived after a fifth embryo transfer. Her history included two prior laparoscopies for suspected endometriosis, performed one and two years earlier, due to infertility and chronic lower abdominal pain. During the first operation, a few superficial endometriotic lesions on the right uterosacral ligament were coagulated. Scarring was described throughout the rectouterine pouch without active endometriotic lesions. During the second laparoscopy one year later, multiple endometriotic deposits were noted on the posterior uterine wall. Superficial lesions were coagulated and the peritoneum was stripped from both ovaries, the parametria, the left vesicouterine fold, and bilaterally in the pararectal region of the pouch of Douglas. A deep infiltrating endometriosis lesion on the right uterosacral ligament was partially resected.

During the first stage of labor, the bleeding stopped, and an oxytocin infusion was started at 4 cm cervical dilation. At 5 cm cervical dilation, meconium-stained amniotic fluid was noted, and the patient reported a sudden onset of severe abdominal pain accompanied by continuous uterine contractions. The oxytocin infusion was immediately stopped, and hexoprenaline (2 ml) was administered. Despite this, the abdominal pain persisted. Ultrasonography revealed no evidence of placental abruption or overt uterine rupture; there was no vaginal bleeding, and the fetal heart rate remained normal. At that time, the cervix was fully dilated, and the fetal head was at +1 station.

**Fig. 1:**
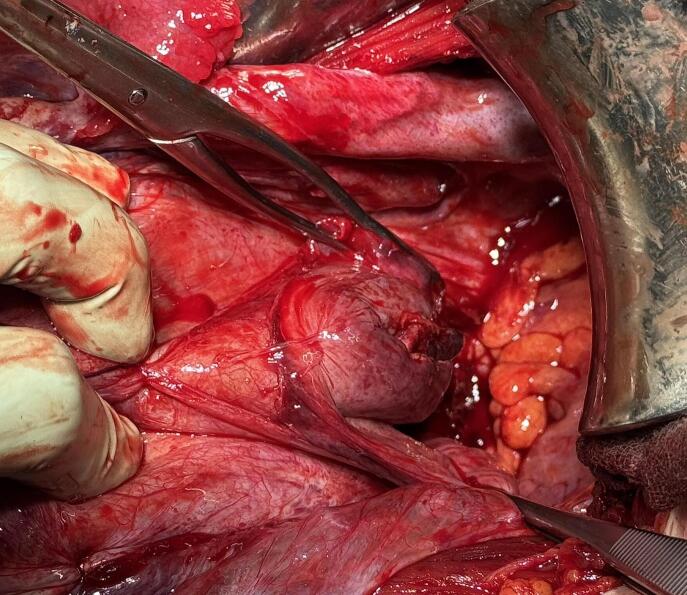
Intraoperative image of the 12 cm longitudinal rupture of the posterior uterine wall extending into the pouch of Douglas, with associated involvement of the posterior vaginal fornix.

Due to persistent, uncontrollable pain, the decision was made to proceed to emergency cesarean section. Upon entering the abdomen and opening the parietal peritoneum, a small amount of amniotic fluid containing meconium was noted. No intra-abdominal bleeding was observed. The anterior uterine wall and fundus were intact. Delivery of the fetus took approximately four minutes (Apgar scores were 1, 7 and 8, arterial pH 7.13). A longitudinal rupture of the posterior uterine wall, about 12 cm in length, was identified, extending into the pouch of Douglas and involving an opening of the posterior vaginal fornix (Fig. 1). The rupture was repaired with a single-layer suture. After intra-abdominal repair, a vaginal examination was performed. The cervix appeared normal, and the posterior vaginal fornix was confirmed to be securely closed from the abdominal approach. The estimated intraoperative blood loss was 700 ml. The neonate did not require admission to a neonatal or intensive care unit. No abnormalities were observed during six-week postpartum follow-up.

The mother received intravenous antibiotic therapy with co-amoxicillin 24 h postoperatively, followed by oral administration for a further four days. On the second postoperative day, the patient complained of diffuse abdominal pain with marked abdominal distension. A CT scan showed markedly dilated bowel loops, interpreted as Ogilvie syndrome. Pharmacological stimulation of gastrointestinal motility was initiated and resulted in significant improvement on the same day. The patient was discharged home on the fourth postoperative day in good general condition. At the postpartum follow-up five weeks later, the patient showed a physiological course. The patient was informed that an interval of at least 12 months should be observed before the next pregnancy and that a primary cesarean section would be recommended in a future pregnancy.

## 3 Discussion

In this case, uterine rupture occurred despite the absence of uterine scarring from any prior cesarean section or myomectomy, which are considered major risk factors [2], [3], [4], [6], [7]. A particular feature of this rare case is the presence of endometriosis and presumed adenomyosis. A few cases have been reported in which ectopic endometrial tissue was considered the cause of uterine rupture [8], [9], [10], [11], [12]. One report presents a notable case of a patient without previous uterine surgery who suffered an unprovoked uterine rupture in the second trimester associated with extensive diffuse adenomyosis [13]. Another report describes how, during a cesarean section, the uterine tissue was found to be so altered by adenomyosis that a subsequent hysterectomy was the only option [14]. The literature discusses potential mechanisms leading to uterine rupture in the context of ectopic endometrial tissue. One potential cause of myometrial damage in adenomyosis may be the decidualization of adenomyotic tissue during pregnancy, which can lead to a loss of structural integrity in the smooth muscle cells [13].

Operations performed for endometriosis may also be considered as a contributing factor [9], [15]. Specifically, surgical resection of deeply infiltrating rectovaginal endometriosis has been reported in the limited literature as a risk factor for uterine rupture. Some authors have even suggested that the posterior uterine wall should be evaluated by postoperative imaging and before conception following resection of deeply infiltrating endometriosis [16]. One report describes a uterine rupture similar to that in the present case, with a longitudinal tear of the posterior wall extending to the posterior fornix, which was attributed to prior surgery for endometriosis involving excision of a rectovaginal lesion and a lesion on the right uterosacral ligament [17]. It is often challenging to discern the relative contribution of previous endometriosis surgeries and the endometriosis itself to uterine rupture, as has been reported not only in the present case [2], [18].

Various additional risk factors are described that are not as strongly associated with uterine rupture as a previous cesarean section or myomectomy, but which should nonetheless be considered when analyzing this case. An increased risk of uterine rupture is associated with labor induction using prostaglandins, labor augmentation with oxytocin, antepartum fetal death, previous first-trimester miscarriages, multiparity, fetal macrosomia, fetal malpresentation, and placenta accreta spectrum [2], [7], [19], [20]. None of these risk factors were present in this case, apart from the use of oxytocin. The use of oxytocin, pre-existing endometriosis and presumed adenomyosis, as well as previous surgical resection stand out as risk factors in this case. It remains unclear whether ectopic endometrial tissue alone, through structural weakening of the posterior uterine wall, was responsible for the rupture, or whether previous surgical interventions involving peritoneal stripping near the uterus also contributed to the defect. This case highlights the importance of considering endometriosis, including previous surgeries for its treatment, as a risk factor for the rare event of uterine rupture in an unscarred uterus.

## 4 Conclusions

This case report describes a rare occurrence in which endometriosis appears to have played a causal role in uterine rupture, underscoring the importance of recognizing endometriosis and its treatment as potential risk factors.

## Contributors

Julia Häusler contributed to patient care, conception of the case report, acquiring and interpreting the data, drafting the manuscript and undertaking the literature review.

Claudia Studer contributed to patient care, conception of the case report, interpreting the data, drafting the manuscript and revising the article critically for important intellectual content.

Kathrin Bütikofer contributed to interpreting the data and revising the article critically for important intellectual content.

Nebojša Stevanović contributed to patient care, conception of the case report and revising the article critically for important intellectual content.

All authors approved the final submitted manuscript.

## Patient consent

Written informed consent was obtained from the patient for publication of the case report and accompanying image.

## Provenance and peer review

This article was not commissioned and was peer reviewed.

## Funding

No funding from an external source supported the publication of this case report.

## Declaration of competing interest

The authors declare that they have no competing interest regarding the publication of this case report.
